# Gastric Cancer Maximum Tumour Diameter Reduction Rate at CT Examination as a Radiological Index for Predicting Histopathological Regression after Neoadjuvant Treatment: A Multicentre GIRCG Study

**DOI:** 10.1155/2018/1794524

**Published:** 2018-03-15

**Authors:** Maria Antonietta Mazzei, Giulio Bagnacci, Francesco Gentili, Andrea Nigri, Veronica Pelini, Carla Vindigni, Francesco Giuseppe Mazzei, Gian Luca Baiocchi, Frida Pittiani, Paolo Morgagni, Enrico Petrella, Gianni Mura, Beatrice Verdelli, Maria Bencivenga, Simone Giacopuzzi, Daniele Marrelli, Franco Roviello, Luca Volterrani

**Affiliations:** ^1^Department of Medical, Surgical and Neuro Sciences, Unit of Diagnostic Imaging, Azienda Ospedaliera Universitaria Senese, University of Siena, Siena, Italy; ^2^Faculty of Statistics, Sapienza University of Rome, Roma, Italy; ^3^Department of Molecular and Developmental Medicine, Unit of Pathology, University of Siena, Siena, Italy; ^4^Unit of Diagnostic Imaging, Azienda Ospedaliera Universitaria Senese, Siena, Italy; ^5^Surgical Clinic, Department of Experimental and Clinical Sciences, University of Brescia, Brescia, Italy; ^6^Department of Radiology, ASST Spedali Civili Brescia, Brescia, Italy; ^7^Department of General Surgery, Morgagni-Pierantoni Hospital, Forlì, Italy; ^8^Radiology Unit, Morgagni-Pierantoni Hospital, Forlì, Italy; ^9^Department of Surgery, Valdarno Hospital, Arezzo, Italy; ^10^Department of Radiology, Valdarno Hospital, Arezzo, Italy; ^11^General and Upper GI Surgery Division, Department of Surgery, University of Verona, Verona, Italy; ^12^Department of Medical, Surgical and Neuro Sciences, Section of Surgery, Azienda Ospedaliera Universitaria Senese, University of Siena, Siena, Italy

## Abstract

**Aim:**

To investigate the role of maximum tumour diameter (D-max) reduction rate at CT examination in predicting histopathological tumour regression grade (TRG according to the Becker grade), after neoadjuvant chemotherapy (NAC), in patients with resectable advanced gastric cancer (AGC).

**Materials and Methods:**

Eighty-six patients (53 M, mean age 62.1 years) with resectable AGC (≥T3 or N+), treated with NAC and radical surgery, were enrolled from 5 centres of the Italian Research Group for Gastric Cancer (GIRCG). Staging and restaging CT and histological results were retrospectively reviewed. CT examinations were contrast enhanced, and the stomach was previously distended. The D-max was measured using 2D software and compared with Becker TRG. Statistical data were obtained using “R” software.

**Results:**

The interobserver agreement was good/very good. Becker TRG was predicted by CT with a sensitivity and specificity, respectively, of 97.3% and 90.9% for Becker 1 (D-max reduction rate > 65.1%), 76.4% and 80% for Becker 3 (D-max reduction rate < 29.9%), and 70.8% and 83.9% for Becker 2. Correlation between radiological and histological D-max measurements was strongly confirmed by the correlation index (c.i.= 0.829).

**Conclusions:**

D-max reduction rate in AGC patients may be helpful as a simple and reproducible radiological index in predicting TRG after NAC.

## 1. Introduction

In the Western world, almost two-thirds of patients with gastric cancer (GC) have locally advanced tumours (stage IIIA or B, or IV) at the time of diagnosis with a less than 50% chance of radical surgery [1, 2]. For this reason, neoadjuvant chemotherapy (NAC) has been introduced successfully in the treatment of locally advanced gastric adenocarcinoma in the United States and Europe, improving both progression-free survival (PFS) and overall survival (OS) over surgery alone in patients with operable tumours [3–5].

Even if long-term outcomes of patients with advanced gastric cancer (AGC) remain poor despite multimodality treatment [6, 7], patients with clinical response to NAC have a significantly better prognosis than nonresponding patients. For this reason, preoperative identification of responder patients, as well as methods for predicting the outcome of patients submitted to NAC, is crucial in order to provide prognostic information to patients and guide clinicians in further surgical and/or adjuvant treatments [8, 9].

In this sense, histopathological regression (HPR) after chemotherapy is believed to be an important objective parameter of response to therapy and the tumour regression grade (TRG) was first described as a measure of histologic response in gastric cancer patients by Becker et al., who observed histopathological changes in surgical specimens treated with NAC compared with resected cancers from patients treated with surgery alone [10]. The clinical and prognostic values of TRG have been demonstrated in several small studies, and, more recently, it was found to be an independent prognostic factor in a large series of GCs by the same group. In any case, histological response to NAC is shown in just a minority of such GC patients; furthermore, about 30% of patients with an evident HPR of the primary tumour still die due to recurrence. Thus, the histopathological response to chemotherapy is still not clearly defined, and its predictive value and clinical application in GC remain unclear [11–15]. On this basis, there is an unmet need for a possible role of imaging in the prediction of chemotherapy response and/or in the early identification of nonresponder from responder patients, to prevent ineffective and potentially harmful treatment in nonresponders. In addition, emerging imaging methodologies for achieving this goal are not always reproducible and easy to use [8, 16–19].

The purpose of our study is therefore to investigate the feasibility of GC maximum tumour diameter (D-max) reduction rate at CT examination as an effective, easy to use, and reproducible radiological index in predicting TRG after NAC. This multicentre study was conducted by the Italian Research Group for Gastric Cancer (GIRCG), a multidisciplinary research group including clinicians (surgeons, pathologists, gastroenterologists, medical oncologists, radiologists, nutritionists, and statisticians) with recognised expertise in GC diagnosis, care, and research from over 25 specialised centres in Italy.

## 2. Materials and Methods

### 2.1. Patients

This study was approved by the institutional review boards of our hospitals, and written informed consent was obtained from all subjects. Abdominal CT examinations of 86 patients were retrospectively reviewed from a cohort of 103 consecutive patients treated with NAC followed by gastrectomy in 5 GIRCG centres (Siena, Forlì, Montevarchi, Brescia, and Verona), between January 2010 and June 2017. Inclusion criteria consisted of biopsy-proven, locally-advanced GC without distant metastases (i.e., clinical parameters T ≥ 3 and/or N+, M0). Seventeen out of 103 patients were excluded because of an interval between CT and surgery longer than 45 days (*n* = 3), presence of metastatic disease at restaging CT (*n* = 4), neoplastic involvement of the esophagus (*n* = 2), clinical complications during chemotherapy needing urgent surgery (*n* = 3), and inappropriate CT methodology or technical parameters (stomach not distended or slice thickness ≥5 mm, *n* = 5). All the 86 patients included in the study (53 men and 33 women, mean age 62.1 years, range 26.7–78) underwent staging CT examination before NAC and a restaging CT performed within a maximum period of 45 days (mean 26 days, range: 1–45) from surgery; sixteen out of 86 patients also underwent an intermediate CT examination after 2 cycles of chemotherapy (early assessment; CT1 pre-NAC, CT2 intermediate, and CT3 presurgical), totalling to 188 examinations. Different chemotherapy regimens are reported in Table 1.

### 2.2. CT Imaging Protocol

CT scans (CTs) were obtained using a 64-detector row configuration (LightSpeed Plus and VCT, General Electric Healthcare, Milwaukee, USA) for 40 patients, a 16-detector row configuration (LightSpeed 16 Pro, General Electric Healthcare, Milwaukee, USA) for 19 patients, a 32-detector row configuration (VCT, General Electric Healthcare, Milwaukee, USA) for 18 patients, and a 128-detector row configuration (Somatom Definition Flash DE, Siemens) for the remaining 9 patients (Table 2). All patients who underwent the CTs had fasted for 8 hours. CTs, acquired with a spiral technique, were preceded by stomach distension obtained with air or water; the stomach was considered distended when gastric folds appeared mostly flat in the tumour location. To avoid possible bias in measuring tumour D-max, the same technique of stomach inflation was used both in staging and restaging CTs for each patient. Air distension was obtained by administering per os two pouches of effervescent granules, together with 10 ml of water, 3 minutes before the scan, whereas in the second method (water distension), the patient was requested to drink 3 or 4 glasses (125 ml) of water immediately before CT examination. All patients also received 1 mg of glucagone (Glucagen, Novo Nordisk) or 20 mg of hyoscine butylbromide (Buscopan, Pharmamedix) intravenously injected to induce gastric hypotonia. After a scout view, an unenhanced upper abdominal CT scan was acquired from the diaphragmatic domes to 2 cm below the lower margin of the gastric body to confirm the distension of the stomach. Contrast-enhanced CTs were performed in the late arterial phase (start delay 45–50 s) in the upper abdomen and in the portal venous phase (start delay 70–80 s) from the pelvic brim to the thoracic inlet, after an intravenous injection of 2 mL/kg of nonionic contrast material (iodine concentration ≥ 350 mg/ml), followed by 40 mL of saline solution, using a semiautomated power injector (3,5–4 mL/s flow rate) with an 18/20-gauge needle in the antecubital vein. A delayed CT scan after 5 minutes was used to characterise uncertain liver lesions. The CT technical parameters are reported in Table 3. An automatic current modulation tube was used to minimise radiation exposure. A standard reconstruction algorithm was used, and patients were instructed not to breathe during helical imaging to avoid motion artefacts.

### 2.3. Image Analysis

**Figure 1:**
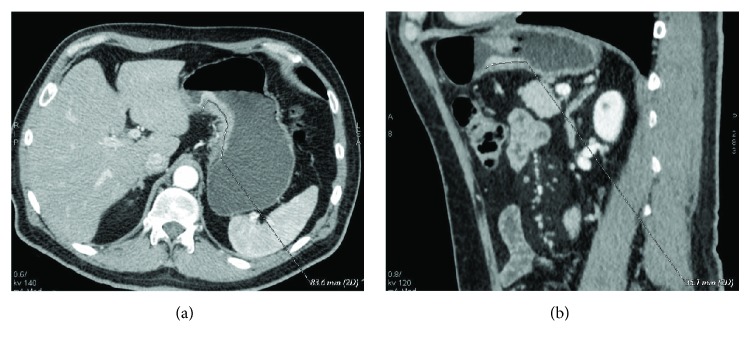
(a, b) Staging (a) and restaging (b) CTs after NAC in a 63-year-old male with a mixed GC. D-max was measured in 2 different planes, respectively, axial oblique in (a), D-max 83.6 mm, and sagittal in (b), D-max 35.1 mm, in order to identify the maximum tumour extension.

All 188 CT examinations (140 for 70 patients and 48 for 16 patients) were analysed in the arterial late phase on a reconstruction and image interpretation console (Advantage Workstation 4.1/3, GE) adjusting the image's level, window and enlargement values each time, and routinely using a 2D multiplanar reconstruction technique (coronal, sagittal, and oblique planes). Images were independently reviewed by two blinded readers, a resident radiologist and a radiologist with 4- and 15-year experience in abdominal CT, respectively (FG and MAM). Readers were asked to review the images of each patient in a random manner, avoiding the date on which they were performed, tumour histotype, and HPR. Maximum tumour diameter (D-max), enhancement of the lesion, and depth of tumour invasion (T parameter) were evaluated. D-max was measured using a curved line through 2D multiplanar reconstructions in order to obtain the maximum tumour extension; all the possible different planes were evaluated for each exam because of the different positioning of the stomach between the different CTs and/or changed orientation of maximum tumour diameter in case of response to NAC (Figure 1); if the lesion was small and ulcerated, D-max was acquired measuring ulcer contour, according to histopathological procedures. Regarding enhancement of the lesion, the ratio between Hounsfield unit (HU) values of the lesion and the aorta in the late arterial phase was calculated to avoid errors arising from small differences in the scan timing from contrast agent injection or dissimilar quality/amount of the contrast agent. For this purpose, a region of interest (ROI), as large as possible with a minimum area of 9 mm^2^, was placed over the lesion and another ROI was placed over the aorta (minimum area of 20 mm^2^). The T parameter was evaluated using all the postcontrast phases available, distinguishing T ≥ 3 from T ≤ 2, according to CT criteria for T staging of the AJCC cancer staging manual (8th edition) and based on the concept that the normal gastric wall is typically seen as a three-layered pattern on the contrast-enhanced CT images [18]. Any disagreement was discussed until a consensus was reached. Finally, the results regarding the D-max and T parameters were compared with the histological data after surgery, in particular, correlating the D-max reduction rate at restaging CT to histological TRG.

### 2.4. Surgical Features

General preoperative indications for treatment were histological diagnosis of GC obtained with upper digestive endoscopy and biopsy and the absence of distant metastases. A staging laparoscopy with cytology on peritoneal washing was performed in 47 out of 86 patients, in order to exclude the presence of peritoneal metastases. The surgical procedure was performed according to GIRCG guidelines [4]. For tumours located in the middle and lower third of the stomach, a subtotal gastrectomy was generally preferred with an adequate proximal resection margin. In all other cases, total gastrectomy with Roux-en-Y reconstruction was the preferred procedure. The gastrectomy was completed by bursectomy and removal of the greater omentum and regional lymph nodes (LNs) [20, 21]. Systematic removal of LN station numbers 7 (left gastric artery), 8a and 8p (common hepatic artery), 9 (celiac artery), 11 (splenic artery), 12a and 12p/b (hepatoduodenal ligament), 13 (retropancreatic), and 14v (superior mesenteric vein) was performed, whereas station 10 (splenic hilum) was removed optionally. With reference to the paraaortic area, after the Kocher manoeuvre, the resection of nodes between the level of the celiac axis and the left renal vein (station 16-a2) and nodes between the left renal vein and the inferior mesenteric artery (station16-b1) was performed optionally [22]. Splenectomy was performed only in the case of direct involvement by the tumour, or in tumours located in the proximal greater curvature. Following surgery, single LNs were retrieved on the fresh specimen by the surgeon and classified in Japanese Research Society for Gastric Cancer (JRSGC) nodal stations for pathological examination [23].

### 2.5. Pathological Analysis

Histopathological findings were evaluated by expert gastrointestinal pathologists. At the macroscopic examination, tumours were classified according to the criteria proposed by Borrmann into polypoid, fungating, ulcerated, and infiltrative [24]. The tumour location was classified as upper, upper/middle, middle, middle/lower, and lower third. The maximum tumour size was performed measuring the residual tumour extension and the scarring area of the precedent tumour (tumour bed), and macroscopic distance of the lesion from the proximal and distal surgical margins was reported.

At least five tissue blocks from the tumour site were taken if tumour was grossly visible; if the viable tumour was not grossly evident, the whole suspicious area was embedded with step sectioning at 5 mm [13]. Antrum and body samples were collected, and proximal and distal resection margins were removed. LNs were distinguished in stations according to the JRSGC classification [23]. Each LN was sectioned on the plane of the largest size. Sections were embedded in paraffin, sectioned at 5 microns, and coloured by hematoxylin and eosin staining.

All the following findings were reported in the microscopic report according to GIRCG guidelines [4]: WHO classification and grading (WHO 2010), Lauren histotype, depth of infiltration, presence or absence of lymphovascular and/or perineural invasion, state of resection margins, chemotherapy-induced alterations, such as replacement of the tumour by fibrous or fibro-inflammatory granulation tissue, histiocytic reaction with hemosiderin-laden and foamy macrophages, acellular mucus lakes, cholesterol deposits, dystrophic calcifications, and vascular changes. Tumour regression grade (TRG) was evaluated according to the Becker classification as percentage of residual neoplasia in the macroscopically evaluated tumour bed. TRG1a was defined as complete tumour regression without residual tumour; TRG1b as subtotal tumour regression with <10% residual tumour cells; TGR2 as partial tumour regression with 10–50% residual tumour cells; and TRG3 as minimal or absent regression with >50% residual tumour cells with or without signs of treatment effects [10, 13, 25].

The total number of examined lymph nodes, total number of positive lymph nodes, and the topography of examined and positive lymph node stations were also reported.

Cancer staging and residual tumour in surgical margins (R) were classified according to AJCC 8th edition [1].

### 2.6. Statistical Analysis

The interobserver agreement was obtained by applying a Kappa test. The Kappa unit ranged from 0 (chance agreement) to 1 (total agreement). In particular, *K* values were deciphered in the following way: *K* < 0.20, poor agreement; *K* = 0.21–0.40, fair; *K* = 0.41–0.60, moderate; *K* = 0.61–0.80, good; and *K* = 0.81–1.00, very good. All the radiological D-max and HU values of gastric lesion and aorta were provided as the average result of measurements of each reviewer from each CT examination. CT results were compared with histological results, in particular lesion size (D-max), TRG, and depth of tumour invasion (T). Statistical analysis was performed using “R” software (multiplatform operative system, GPL licence). Radiological D-max investigation was the focus of the statistical analysis. First of all, the distributions of the D-max values before and after NAC were studied by Shapiro-Wilk test and QQ-plot elaboration, and, given the results, we proceeded with a nonparametric approach; a bootstrap method was used to individuate D-max mean values before and after neoadjuvant chemotherapy and differences were studied graphically and by the Wilcoxon test. A similar analysis was conducted in order to investigate differences in the 16 patients who underwent intermediate CT using the ANOVA test and, for multiple comparisons, *t* test for independent samples and Bonferroni correction. A *p* value < 0.05 was considered statistically significant. D-max was also investigated in terms of reduction rate at restaging CT compared with staging CT (in the entire population and in the population broken down by histotype), and the correlation between this parameter and TRG was investigated. In particular, patients were divided into 3 groups, analogously to the Becker classification, through two successive dichotomous divisions, and the optimal cutoff values were determined through ROC curves analysis. Lesion enhancement analysis was performed calculating the ratio between gastric lesion and aorta HU; the ratio between normalised values obtained before and after NAC was calculated and plotted versus different Becker grades. Differences between radiological and pathological measurements of D-max were investigated through nonparametric regression and Bland-Altman plot, and the exact identification of T parameter (T ≤ 2 or T ≥ 3) was expressed in terms of sensitivity, specificity, VPP, VPN, and accuracy.

## 3. Results

**Figure 2:**
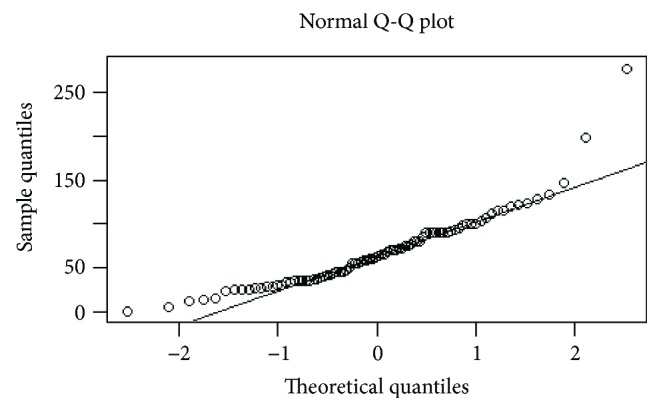
Distribution of D-max values before neoadjuvant chemotherapy: QQ plot demonstrates a nonnormal distribution.

**Figure 3:**
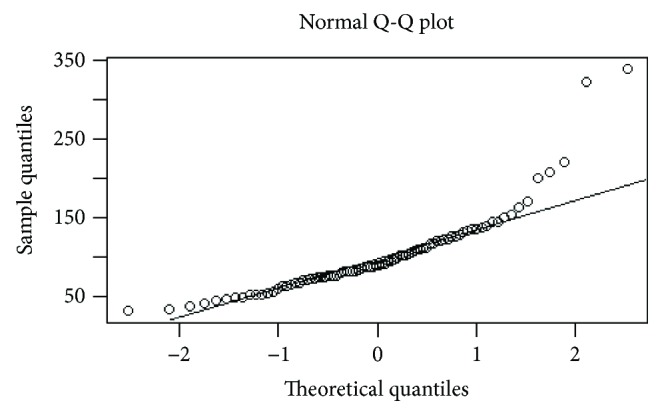
Distribution of D-max values just before surgery: QQ plot demonstrates a nonnormal distribution.

**Figure 4:**
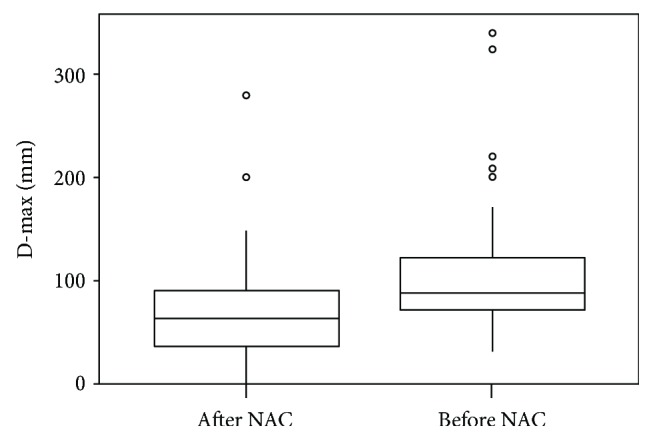
Box plot of D-max values before and after NAC.

**Figure 5:**
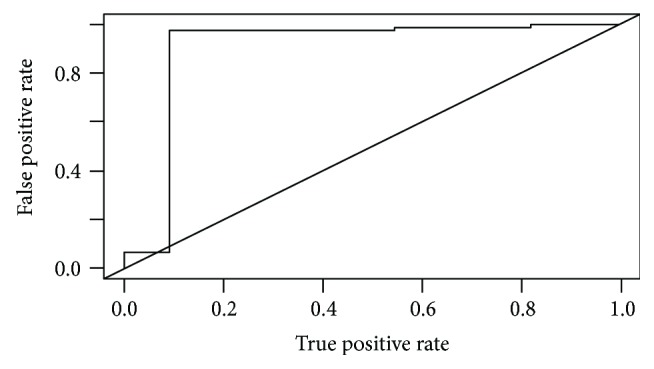
ROC curve of D-max reduction rate to predict Becker grade 1.

**Figure 6:**
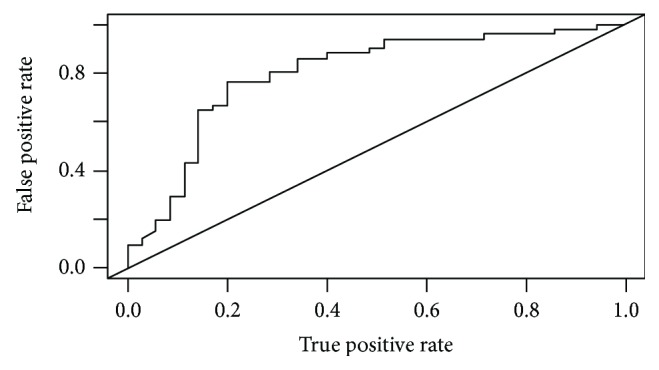
ROC curve of D-max reduction rate to predict Becker grade 3.

**Figure 7:**
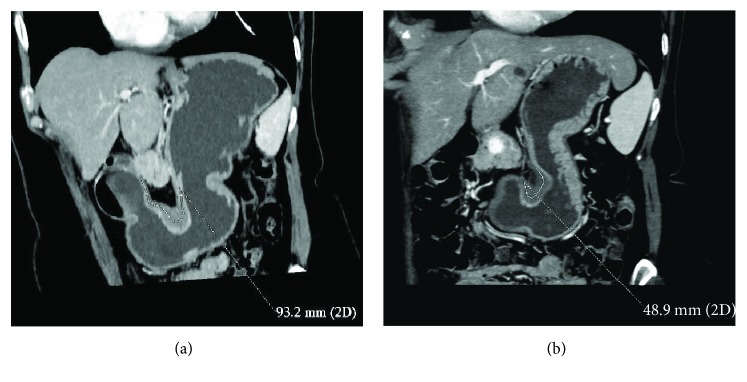
(a, b) GC cancer of the angulus (diffuse histotype) in a 69-year-old woman. TRG 2 was found on final pathology after NAC. Tumour D-max reduction rate (47.5%), between CT before (a) and after NAC (b), correctly identified TRG.

At histopathology, according to the Becker et al. classification, 11 patients (12.8%) had a TRG 1, 24 (27.9%) a TRG 2, and 51 (59.3%) a TRG 3, whereas according to the AJCC classification, 31 (36%) patients resulted in T ≤ 2 and 55 (64%) T ≥ 3. Mean maximum histopathological tumour diameter was 53.7 mm. There was a prevalence of the Lauren intestinal histotype with 43 intestinal (53%), 34 diffuse (40%), 7 mixed (8%), and 2 nonclassifiable (2%) tumours. A good/very good agreement (0.77-1) was found between the two readers concerning CT findings, and the patients with T ≥ 3 at histopathological examination were individuated by CT with a sensibility of 92.73%, specificity of 64.52%, PPV of 82.26%, NPV of 83.33, and total accuracy of 82.56%. D-max from staging and restaging CT examinations did not demonstrate a normal distribution at the Shapiro-Wilk test (*p* < 0.05) as reported in QQ plots (Figures 2 and 3) thus, in order to avoid statistical errors, mean value distribution was calculated through the bootstrap method resulting in 100.4 mm before and 68.4 mm after NAC, respectively. D-max box plots graphically showed a difference between the 2 groups, before and after NAC (Figure 4), which was then confirmed applying the Wilcoxon test for paired samples (*p* = 1938 e-06, statistically significant), demonstrating that chemotherapy induced a reduction in CT-measured D-max. D-max reduction rate at restaging CT was not significantly different between intestinal and diffuse histotypes (resp., 66% and 75%, *p* > 0.05). The optimal cutoff values of D-max reduction rate at restaging CT, obtained by ROC curve analysis to identify 3 groups of patients, analogously to the Becker classification, were ≥65.1% (corresponding to TRG 1, <10% residual tumour cells, with a sensitivity and specificity of 97.3% 90.9%, resp.) and ≤29.9% (corresponding to TRG 3, >50% of residual tumour cells, with a sensitivity and specificity of 76.4% and 80.0%, resp.) (Figures 5 and 6). Using the same cutoff values reported above, Becker grade 2 patients were predicted with a sensitivity and specificity of 70.8% and 83.9%, respectively (Figure 7).

**Figure 8:**
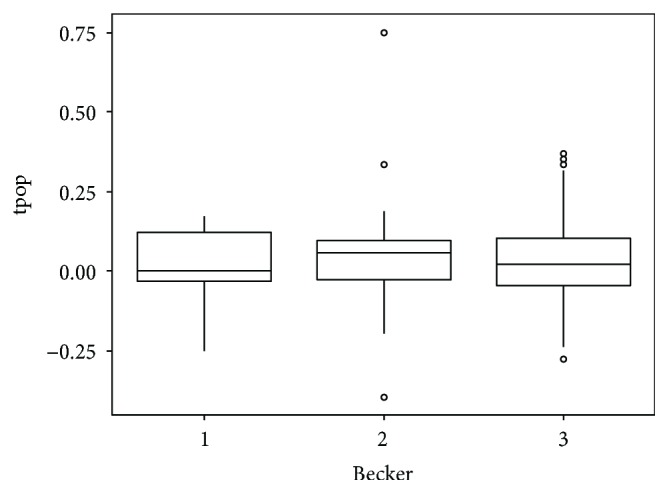
Box plot showing Hounsfield unit (HU) analysis. HUs were normalised through the ratio between the lesion and aorta HU. Values in the *y*-axis were obtained from the ratio between HU before and after NAC, whereas values in the *x*-axis show the Becker grade.

**Figure 9:**
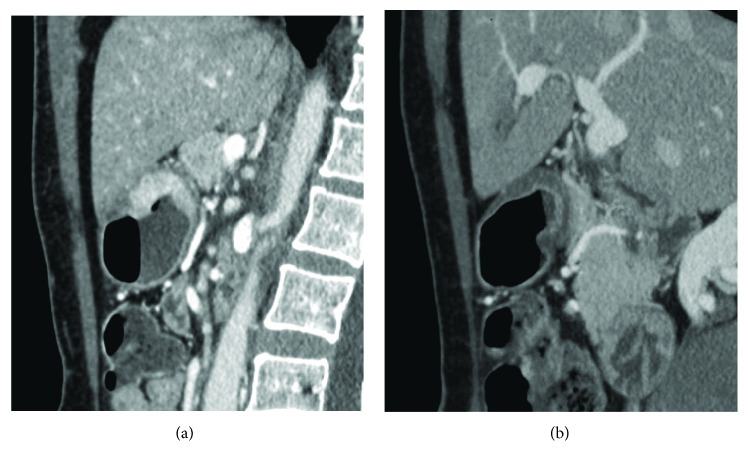
(a, b) A 65-year-old man with GC of the antrum (diffuse histotype). After NAC (b), lesion enhancement is significantly lower than before (a), whereas D-max is substantially unchanged. Patient was correctly classified as TRG 3 on final pathology.

**Figure 10:**
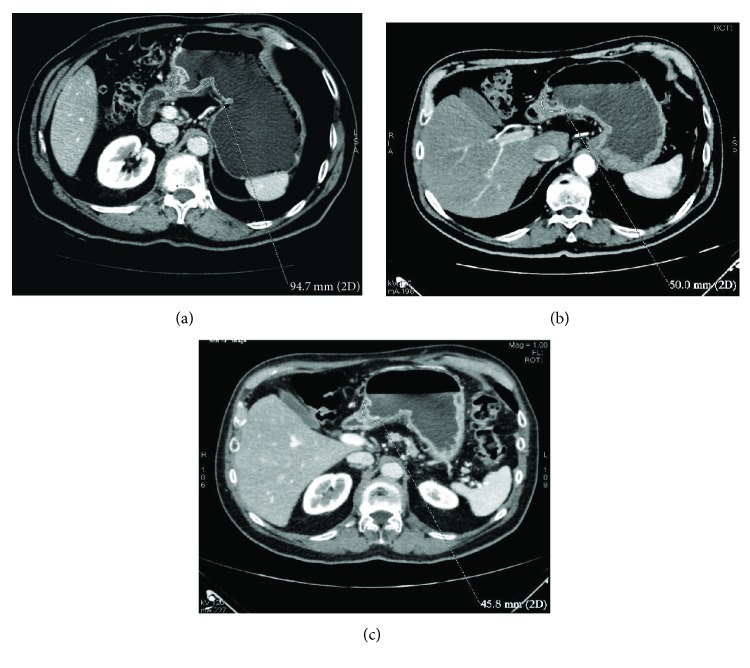
(a–c) Comparison between staging CT (a), intermediate (b) and presurgical (c) CTs in a 71-year-old man with GC (intestinal histotype) located in the antrum and body, along the lesser curvature. It was clear how D-max reduction rate was greater after 2 cycles of chemotherapy ((a) versus (b), 47.2%) than in the remainder of the treatment ((b) versus (c), 10%).

Lesion enhancement analysis before and after NAC did not statistically show significant differences in patients with different Becker grades as shown in the box plots and figures (Figures 8 and 9). Moreover, D-max reduction rate differences were investigated in patients who underwent an intermediate CT after 2 cycles of chemotherapy. The comparison between the D-max reduction rate between CT1 and CT2 (pre-NAC and intermediate CTs, resp.) and between CT2 and CT3 (intermediate and pre-surgical CTs, resp.) resulted statistically different when applying both the ANOVA test and post hoc Student's *t*-test (*p* = 0.00057 between CT1 and CT2 while *p* = 0.02 between CT2 and CT3) suggesting that the major tumour regression occurred after 2 cycles of chemotherapy (Figure 10).

**Figure 11:**
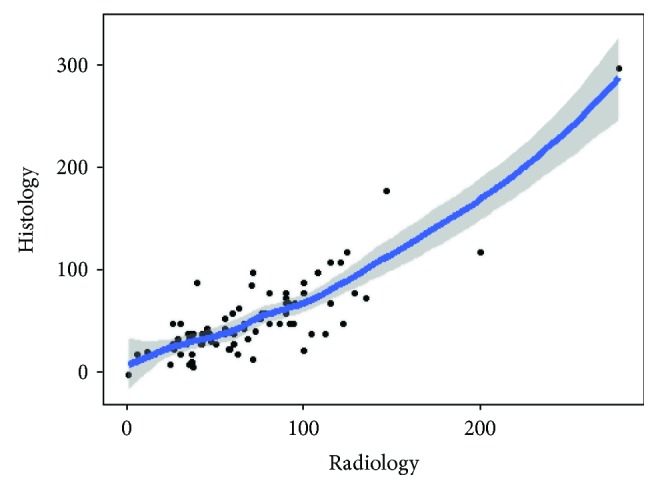
Nonparametric regression; histological versus radiological D-max measurements.

**Figure 12:**
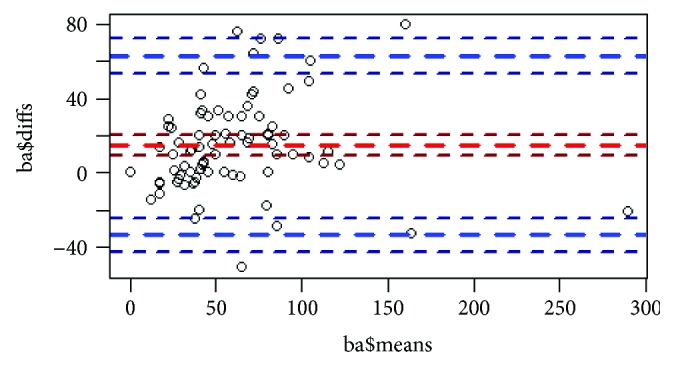
Bland-Altman plot: the casual distribution of the points demonstrates that radiological and pathological D-max measurement methods are theoretically interchangeable.

Finally, correlation between radiological and pathological D-max measurement was graphically investigated through nonparametric regression (Figure 11) and strongly confirmed by the correlation index (c.i. = 0.829); the Bland-Altman plot confirmed a high agreement between radiological and pathological D-max measurement methods (Figure 12).

## 4. Discussion

HPR after NAC has been shown to be an important objective parameter of improved survival in a high percentage of patients affected by GC [13, 25]. Thus, in this severe disease, frequently diagnosed in an advanced stage and with poor prognosis, an accurate assessment of tumour response to chemotherapy is fundamental in treatment decision-making [26].

In particular, it would be worth identifying responder patients, prior to surgery, who may benefit from aggressive multimodal treatments, achieving patient-centred care and better health care cost management in this way. Nowadays, the majority of clinical trials evaluating response to cancer treatment by imaging use the response evaluation criteria in solid tumours (RECIST) [27, 28]; however, GC lesion is defined as nonmeasurable by RECIST, given that the stomach is not a parenchymatous organ [29]. Moreover, RECIST response criteria seem to underestimate histological gastric tumour response [26], so their implementation is poor. The role of metabolic imaging, like PET, in this field has been investigated in the literature since 2003; one of the first studies by Ott et al. [16], which analysed 44 patients, showed the potential value of this technique in monitoring the efficacy of NAC in gastric cancer, predicting histopathological response in 77% of responders and 86% of nonresponders; however, several later studies did not confirm these results, and, in fact, it was demonstrated how the percentage change in maximum SUV did not significantly correlate with the grade of HPR [8, 30]. Moreover, not all gastric tumours, in particular diffuse histotype and tumour containing mucus, show FDG uptake thus the results of different series are influenced by a percentage of different histotypes included in the study population [31]. Compared with PET imaging, CT is not influenced by the limit of the uptake tracer and at the same time allows a morphological and functional evaluation, the latter through contrast agent administration, even if the correct methodology and choice of technical parameters in performing the exam are essential prerequisites for obtaining reliable data: in particular gastric distension of the stomach, a thin effective slice thickness and tube voltage/reference mAs appropriated to patient size [32–34]. In this regard, Yoshikawa et al. reported poor overall accuracy in determining T parameter after NAC, suggesting that T staging by CT should not be considered in clinical decision-making [35], but they performed CT scans using a slice thickness of 5–7 mm which is not acceptable, considering the CT technology currently available. On the other hand, Lee et al. evaluated tumour response to NAC through CT, analysing the volume reduction rate of gastric cancer, which was found to be significantly correlated to HPR [8], even if whole tumour volume has to be extracted manually tracing lesion boundaries for each slice, a process that is extremely laborious and hardly applicable in daily clinical practice. The aim of our study was to investigate the utility of an easily applicable radiological index (D-max) at CT examinations in predicting HPR after NAC in GC, and our results showed that D-max reduction rate seems to be reliable for identifying responder patients, in particular Becker/TRG 1 patients. Furthermore, the greater D-Max percentage reduction after 2 cycles of NAC in patients who underwent an intermediate CT is an interesting point which may suggest anticipating surgery in responder patients; this result should certainly be confirmed in larger series study even if some suggestions in this sense are already reported in the literature [36]. More evidence could come from the survival results of the ongoing randomised phase-II study by GIRCG, comparing a preoperative versus perioperative (pre- + post-operative) docetaxel, oxaliplatin, and capecitabin (DOX) regimens in patients with locally advanced resectable GC.

Gastric lesion enhancement analysis did not show statistically significant results for predicting HPR. For that reason, even an important lesion contrast uptake reduction after NAC should not be considered highly suggestive of tumour response. This data clearly contrasts with those of Liu et al. who adopted HU analysis to evaluate tumour response according to adapted Choi criteria; since their results were correlated to PFS and OS, long-term follow-up of our patients is needed to draw conclusions in this regard [26].

Some limitations of our study should be noted. First, it is a retrospective study, although all CT examinations were reevaluated in a prospective setting. Second, the examination technique changed during the study period (5 different CT scanners with slightly different examination parameters and little variation of delay scan after contrast material injection). Third, there is a lack of patient subdivision on the basis of different chemotherapy regimens and there is a small number of patients. Fourth, in the case of large-sized lesion, it can be difficult to measure radiological D-max in a reproducible way despite 2D multiplanar reconstructions. Finally, the HPR of gastric tumour was not related to the HPR of lymph nodes, which would be very important, in particular in T2 tumours. While CT global accuracy on the N parameter reaches about 90% at staging, adopting a double size cutoff (5 mm for perigastric nodes and 8 mm for extraperigastric nodes), at present, there are no established criteria with high accuracy for defining lymph node response to NAC, such as a cutoff size or rate of size reduction; therefore, given the complexity of the issue, further studies are needed in this field, probably differentiating among different tumour histotypes [20]. However, preliminary previous studies have demonstrated that lymph node HPR seems to be closely related with those of primary tumours [37].

In conclusion, our results support the use of CT in evaluating gastric cancer response to NAC on the condition that CT examination is performed using a dedicated protocol and images are analysed by an experienced reader. A prospective, multicentric GIRCG trial is ongoing in order to obtain definitive results.

## Figures and Tables

**Table 1:** Different chemotherapy regimens of all patients.

Chemotherapy regimen	Patients (number)
ECF (epirubicin, cisplatin, and 5-fluorouracil)	16
DOX (docetaxel, oxaliplatin, and capecitabine)	46
EOX (capecitabine, oxaliplatin, and ED epirubicin)	7
DCF (docetaxel, cisplatin, and 5-fluorouracil)	2
2ECF + 4DCF	1
CDDP(cisplatin) + capecitabine	1
FOLFOX (folic acid, fluorouracil, and oxaliplatin)	9
1 DOX + 3 DOF (docetaxel, oxaliplatin, and fluorouracil)	1
EOF (epirubicin, oxaliplatin, and 5-fluorouracil)	3

**Table 2:** List of tomographs adopted in the study.

GIRCG center	Tomograph	Layers	Patients (number)
Siena	VCT, GE Healthcare	64	32
Forlì	LightSpeed Pro, GE Healthcare	16	19
Montevarchi	LightSpeed Pro, GE Healthcare	32	18
Brescia	Siemens Somatom Definition Flash DE	128	9
Verona	LightSpeed, GE HealthCare	64	8

**Table 3:** CT technical parameters. Slice thickness is reported for the late arterial phase.

CT technical parameters	
Slice thickness (mm)	Patients (number)
1.25	30
2	20
2.5	17
3.75	8
Beam pitch	0.9/1.3
Reconstruction interval (IR)	At least half of the effective slice thickness
Tube voltage (kVp)	120–140
Reference mAs	200/250–500/600

## References

[1] Amin M. B., Edge S., Greene F. (2017). *AJCC Cancer Staging Manual*.

[2] Roder J. D., Böttcher K., Siewert J. R. (1993). Prognostic factors in gastric carcinoma. Results of the German gastric carcinoma study 1992. *Cancer*.

[3] Cunningham D., Allum W. H., Stenning S. P. (2006). Perioperative chemotherapy versus surgery alone for resectable gastroesophageal cancer. *The New England Journal of Medicine*.

[4] De Manzoni G., Marrelli D., Baiocchi G. L. (2017). The Italian Research Group for Gastric Cancer (GIRCG) guidelines for gastric cancer staging and treatment: 2015. *Gastric Cancer*.

[5] Ychou M., Boige V., Pignon J. P. (2011). Perioperative chemotherapy compared with surgery alone for resectable gastroesophageal adenocarcinoma: an FNCLCC and FFCD multicenter phase III trial. *Journal of Clinical Oncology*.

[6] Smalley S. R., Benedetti J. K., Haller D. G. (2012). Updated analysis of SWOG-directed intergroup study 0116: a phase III trial of adjuvant radiochemotherapy versus observation after curative gastric cancer resection. *Journal of Clinical Oncology*.

[7] Marrelli D., Polom K., de Manzoni G., Morgagni P., Baiocchi G. L., Roviello F. (2015). Multimodal treatment of gastric cancer in the west: where are we going?. *World Journal of Gastroenterology*.

[8] Lee S. M., Kim S. H., Lee J. M. (2009). Usefulness of CT volumetry for primary gastric lesions in predicting pathologic response to neoadjuvant chemotherapy in advanced gastric cancer. *Abdominal Imaging*.

[9] Gruppo Italiano Ricerca Cancro Gastrico (GIRCG), Chiari D., Orsenigo E. (2017). Effect of neoadjuvant chemotherapy on HER-2 expression in surgically treated gastric and oesophagogastric junction carcinoma: a multicentre Italian study. *Updates in Surgery*.

[10] Becker K., Mueller J. D., Schulmacher C. (2003). Histomorphology and grading of regression in gastric carcinoma treated with neoadjuvant chemotherapy. *Cancer*.

[11] Napieralski R., Ott K., Kremer M. (2005). Combined *GADD45A* and *thymidine phosphorylase* expression levels predict response and survival of neoadjuvant-treated gastric cancer patients. *Clinical Cancer Research*.

[12] Ott K., Lordick F., Becker K. (2008). Glutathione-S-transferase P1, T1 and M1 genetic polymorphisms in neoadjuvant-treated locally advanced gastric cancer: GSTM1-present genotype is associated with better prognosis in completely resected patients. *International Journal of Colorectal Disease*.

[13] Becker K., Langer R., Reim D. (2011). Significance of histopathological tumor regression after neoadjuvant chemotherapy in gastric adenocarcinomas: a summary of 480 cases. *Annals of Surgery*.

[14] Blackham A. U., Greenleaf E., Yamamoto M. (2016). Tumor regression grade in gastric cancer: predictors and impact on outcome. *Journal of Surgical Oncology*.

[15] Schmidt T., Sicic L., Blank S. (2014). Prognostic value of histopathological regression in 850 neoadjuvantly treated oesophagogastric adenocarcinomas. *British Journal of Cancer*.

[16] Ott K., Fink U., Becker K. (2003). Prediction of response to preoperative chemotherapy in gastric carcinoma by metabolic imaging: results of a prospective trial. *Journal of Clinical Oncology*.

[17] Beer A. J., Wieder H. A., Lordick F. (2006). Adenocarcinomas of esophagogastric junction: multi–detector row CT to evaluate early response to neoadjuvant chemotherapy. *Radiology*.

[18] Kim J. W., Shin S. S., Heo S. H. (2012). Diagnostic performance of 64-section CT using CT gastrography in preoperative T staging of gastric cancer according to 7th edition of AJCC cancer staging manual. *European Radiology*.

[19] Mazzei M. A., Preda L., Cianfoni A., Volterrani L. (2015). CT perfusion: technical developments and current and future applications. *BioMed Research International*.

[20] Marrelli D., Mazzei M. A., Pedrazzani C. (2011). High accuracy of multislices computed tomography (MSCT) for para-aortic lymph node metastases from gastric cancer: a prospective single-center study. *Annals of Surgical Oncology*.

[21] Marrelli D., Mazzei M. A., Roviello F. (2014). Gastric cancer with para-aortic lymph node metastases: do not miss a chance of cure!. *Cancer Chemotherapy and Pharmacology*.

[22] Marrelli D., Ferrara F., Giacopuzzi S. (2017). Incidence and prognostic value of metastases to “posterior” and para-aortic lymph nodes in resectable gastric cancer. *Annals of Surgical Oncology*.

[23] Japanese Gastric Cancer Association (2011). Japanese classification of gastric carcinoma: 3rd English edition. *Gastric Cancer*.

[24] Borrmann R., Henke F., Lubarsch O. (1926). *Handbuch der Speziellen Patologischen Anatomie und Histologie*.

[25] Becker K., Reim D., Novotny A. (2012). Proposal for a multifactorial prognostic score that accurately classifies 3 groups of gastric carcinoma patients with different outcomes after neoadjuvant chemotherapy and surgery. *Annals of Surgery*.

[26] Liu K., Li G., Fan C., Zhou C., Li J. (2012). Adapted Choi response criteria for prediction of clinical outcome in locally advanced gastric cancer patients following preoperative chemotherapy. *Acta Radiologica*.

[27] Kwee R. M., Kwee T. C. (2014). Role of imaging in predicting response to neoadjuvant chemotherapy in gastric cancer. *World Journal of Gastroenterology*.

[28] Eisenhauer E. A., Therasse P., Bogaerts J. (2009). New response evaluation criteria in solid tumours: revised RECIST guideline (version 1.1). *European Journal of Cancer*.

[29] Kurokawa Y., Shibata T., Sasako M. (2014). Validity of response assessment criteria in neoadjuvant chemotherapy for gastric cancer (JCOG0507-A). *Gastric Cancer*.

[30] Vallböhmer D., Hölscher A. H., Schneider P. M. (2010). [^18^F]-fluorodeoxyglucose-positron emission tomography for the assessment of histopathologic response and prognosis after completion of neoadjuvant chemotherapy in gastric cancer. *Journal of Surgical Oncology*.

[31] Stahl A., Ott K., Weber W. (2003). FDG PET imaging of locally advanced gastric carcinomas: correlation with endoscopic and histopathological findings. *European Journal of Nuclear Medicine and Molecular Imaging*.

[32] Grassi R., Pinto A., Mannelli L., Marin D., Mazzei M. A. (2016). New imaging in gastrointestinal tract. *Gastroenterology Research and Practice*.

[33] Mazzei M. A., Khader L., Cirigliano A. (2013). Accuracy of MDCT in the preoperative definition of peritoneal cancer index (PCI) in patients with advanced ovarian cancer who underwent peritonectomy and hyperthermic intraperitoneal chemotherapy (HIPEC). *Abdominal Imaging*.

[34] Mazzei M. A., Volterrani L. (2015). Errors in multidetector row computed tomography. *La Radiologia Medica*.

[35] Yoshikawa T., Tanabe K., Nishikawa K. (2014). Accuracy of CT staging of locally advanced gastric cancer after neoadjuvant chemotherapy: cohort evaluation within a randomized phase II study. *Annals of Surgical Oncology*.

[36] Yoshikawa T., Morita S., Tanabe K. (2016). Survival results of a randomised two-by-two factorial phase II trial comparing neoadjuvant chemotherapy with two and four courses of S-1 plus cisplatin (SC) and paclitaxel plus cisplatin (PC) followed by D2 gastrectomy for resectable advanced gastric cancer. *European Journal of Cancer*.

[37] Kinoshita O., Ichikawa D., Ichijo Y. (2015). Histological evaluation for chemotherapeutic responses of metastatic lymph nodes in gastric cancer. *World Journal of Gastroenterology*.

